# Threshold effects of insulin resistance on cognitive function in coronary heart disease: the non-linear diagnostic value of the triglyceride-glucose index

**DOI:** 10.3389/fcvm.2026.1785441

**Published:** 2026-04-14

**Authors:** Shun Zhao, Yejing Zhao, Xiaohong Wu, Changjiang Li, Hongyu Peng

**Affiliations:** Beijing Anzhen Hospital, and Beijing Institute of Heart, Lung and Blood Vessel Diseases, Beijing, China

**Keywords:** cognitive dysfunction, coronary heart disease, insulin resistance, risk stratification, triglyceride-glucose index

## Abstract

**Background:**

Insulin resistance contributes to cognitive dysfunction in patients with coronary heart disease (CHD). We examined the dose-response relationship between the triglyceride-glucose (TyG) index and cognitive status in this population.

**Methods:**

We analyzed data from 839 CHD patients, classifying cognitive function as Normal Cognition, Mild Cognitive Impairment, Severe Cognitive Impairment. Logistic regression and restricted cubic splines modeled the associations. We calculated net reclassification improvement (NRI) and integrated discrimination improvement (IDI) to test the predictive value of TyG beyond standard risk factors.

**Results:**

The severity of cognitive impairment increased with increasing TyG levels. In fully adjusted models, compared to the lowest tertile, the highest TyG tertile was associated with mild cognitive impairment (OR: 1.866, 95% CI: 1.268–2.748) and severe cognitive impairment (OR: 3.255, 95% CI: 1.870–5.668). Spline analysis showed a J-shaped curve for severe cognitive impairment (*P* for non-linearity = 0.029) and a linear trend for mild cognitive impairment (*P* for non-linearity = 0.619). Adding TyG to baseline models improved risk reclassification for severe cognitive impairment (NRI: 0.405, *P* < 0.001; IDI: 0.031, *P* = 0.023) and mild cognitive impairment (NRI: 0.185, *P* = 0.024).

**Conclusion:**

In patients with CHD, the TyG index identifies a specific metabolic threshold associated with severe cognitive impairment. The distinct non-linear trajectory and significant reclassification improvement suggest that the TyG index captures residual metabolic-inflammatory risk not addressed by traditional factors, highlighting its utility as a target for neuroprotective strategies.

## Highlights

**• What is currently known about this topic?**

Insulin resistance contributes to cognitive dysfunction. While TyG serves as a surrogate marker, its specific association with cognitive impairment severity remains unclear.

**• What is the key research question?**

To determine the dose-response relationship between the TyG index and cognitive impairment severity in CHD patients and evaluate its incremental predictive value for risk stratification.

**• What is new?**

We identified a distinct J-shaped threshold for severe cognitive impairment vs. a linear trend for mild deficits. Furthermore, the TyG index significantly improved risk reclassification (NRI > 0.40), capturing residual metabolic-inflammatory risk beyond standard cardiovascular models.

**• How might this study influence clinical practice?**

The TyG index serves to identify residual metabolic risk for cognitive decline. This supports shifting focus to comprehensive metabolic management, suggesting that targeting insulin resistance may improve neuroprotective outcomes in CHD patients.

## Introduction

Cognitive dysfunction is a frequent complication in patients with coronary heart disease (CHD), independently predicting poor medication adherence and increased mortality risk (1, 2). Accumulating evidence suggests that metabolic dysregulation exacerbates vascular cognitive impairment (3). Insulin resistance (IR) promotes neurodegeneration through chronic neuroinflammation, endothelial dysfunction, and impaired amyloid-beta clearance, even in non-diabetic individuals (4–6). Routine assessment of IR in cardiovascular practice remains challenging. The hyperinsulinemic-euglycemic clamp is invasive, and the homeostasis model assessment of insulin resistance (HOMA-IR) requires insulin quantification, which is not standard in all settings (7, 8). The triglyceride-glucose (TyG) index has recently been validated as a reliable surrogate for IR (9), potentially outperforming HOMA-IR in capturing the dual burden of glucotoxicity and lipotoxicity (10, 11).

Although the association between TyG index and cardiovascular outcomes has been established (12, 13), its specific association with cognitive function in populations with CHD remains unclear. Studying this population is critical because CHD patients are highly vulnerable to cognitive decline driven by chronic cerebral hypoperfusion and residual metabolic risks that standard cardiovascular therapies often fail to address. Prior studies have largely focused on general populations or focused solely on binary outcomes (presence vs. absence of dementia), neglecting the continuous dose-response relationship across varying severities of cognitive deficits (14, 15). Furthermore, it is unknown whether incorporating the TyG index into standard risk models provides statistically significant incremental predictive value for identifying high-risk individuals. This study aimed to determine the association between the TyG index and cognitive status (ranging from mild decline to impairment) in CHD patients and to evaluate its potential utility in improving risk stratification.

## Methods

### Study design and population

**Figure 1:**
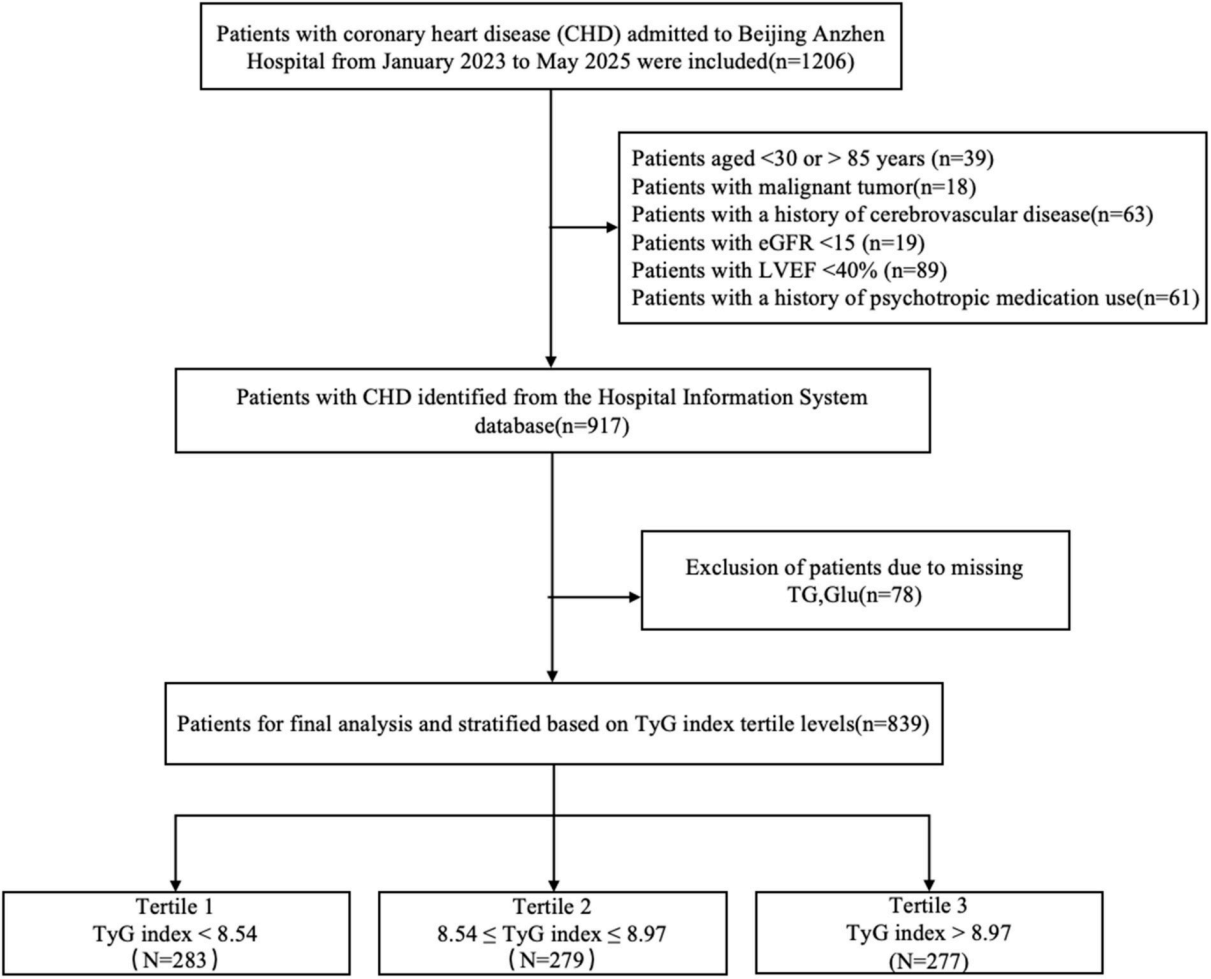
Flowchart of study population selection. The diagram illustrates the inclusion and exclusion criteria applied to the initial cohort of 1,206 patients with CHD, resulting in a final analytical sample of 839 participants. TyG, triglyceride-glucose index.

This was a single-center, cross-sectional study using retrospectively collected data. After applying exclusion criteria to 1,206 consecutive patients, a total of 839 patients with CHD who received treatment at Beijing Anzhen Hospital between January 2023 and May 2025 were finally enrolled (Figure 1). This study was approved by the Ethics Committee of Beijing Anzhen Hospital(No. 2025053). Given its retrospective nature, the requirement for informed consent from patients was waived by the Ethics Committee.

### Data collection and definitions

Clinical data were retrospectively extracted from the Electronic Medical Record system. Baseline variables included demographics, cardiovascular risk factors, and laboratory metrics from fasting venous blood. Echocardiography was recorded to assess cardiac function, primarily evaluated by left ventricular ejection fraction (LVEF). Hypertension was defined as having a resting systolic blood pressure ≥140 mmHg and/or a diastolic blood pressure ≥90 mmHg, or the current use of antihypertensive medications. Diabetes was defined as having a fasting blood glucose ≥7.0 mmol/L, a self-reported history of diabetes, or the current use of glucose-lowering drugs. Smoking and alcohol consumption were categorized as binary variables (current active user vs. non-user) based on their status at admission. A history of cerebrovascular disease was defined as documented prior ischemic stroke or intracerebral hemorrhage.

TyG index was calculated as ln [TG (mg/dL) × glucose (mg/dL)/2].

Cognitive function was evaluated using the Mini-Mental State Examination (MMSE) and the Montreal Cognitive Assessment (MoCA). To account for education levels, validated cut-off scores for the Chinese population were applied. For MMSE, impairment was defined as ≤17 for illiterate patients, ≤20 for those with 1–6 years of education, and ≤24 for those with >6 years of education. For MoCA, one point was added for patients with ≤12 years of education, with a score <26 indicating impairment (16, 17). Given the differing sensitivity profiles of the two screening instruments, we employed a concordance-based severity grading system. The MoCA is recognized for its high sensitivity in detecting mild cognitive impairment (MCI) and dysexecutive syndrome, which are prevalent in CHD populations, whereas the MMSE is more specific for detecting established global dementia but lacks sensitivity for early vascular deficits (18, 19). Therefore, consistent with a gradient of neurocognitive burden, patients were stratified into:

- Normal Cognition: No impairment on either scale.
- Mild Cognitive Impairment (Discordant Group): Impairment on either MoCA or MMSE (predominantly isolated MoCA impairment), reflecting early-stage or domain-specific deficits potentially amenable to compensation.
- Severe Cognitive Impairment (Concordant Group): Concurrent impairment on both scales, indicating a robust, multi-domain cognitive decline that exceeds the detection threshold of the less sensitive MMSE.

### Statistical analysis

Continuous variables were reported as mean ± standard deviation (SD) or median [interquartile range (IQR)], and categorical variables as frequencies (percentages). Group differences were assessed using ANOVA, Kruskal–Wallis, or Chi-square tests. Multivariable logistic regression models were constructed to calculate odds ratios (ORs) and 95% confidence intervals (CIs) for the association between the TyG index and cognitive outcomes. Model 1 was unadjusted; Model 2 was adjusted for age, sex, and education level; and Model 3 was fully adjusted for age, sex, education, BMI, smoking status, drinking status, hypertension, LDL-C, eGFR, and hs-CRP. Subgroup analyses were further performed stratified by age, sex, education, BMI, smoking, drinking, hypertension, LDL-C, eGFR, and hs-CRP to explore potential interactions. To visualize the potential non-linear dose-response relationship, we modeled the TyG index using restricted cubic splines (RCS) with four knots placed at the 5th, 35th, 65th, and 95th percentiles. The incremental predictive value of the TyG index was evaluated using Receiver Operating Characteristic (ROC) curves, Net Reclassification Improvement (NRI), and Integrated Discrimination Improvement (IDI). ROC curves assessed the overall discriminative ability of the models, NRI quantified the proportion of participants correctly reclassified into more accurate risk estimates, and IDI measured the continuous improvement in predicted probabilities between individuals with and without cognitive impairment. All analyses were performed using R software, with a two-tailed *P* < 0.05 considered statistically significant.

## Results

### Baseline characteristics

As detailed in Table 1, demographic characteristics—including age, sex, and educational attainment—were comparable across the normal cognition, mild cognitive impairment, and severe cognitive impairment groups. Lifestyle factors and the prevalence of traditional comorbidities, such as hypertension and diabetes, also showed no significant intergroup disparities. In contrast, distinct metabolic variances were observed: patients with severe cognitive impairment exhibited the most adverse phenotypic profile, characterized by significantly elevated TyG index, systolic blood pressure, fasting glucose, atherogenic lipids (TG, TC), and inflammatory markers (hs-CRP), alongside compromised renal function (reduced eGFR).

**Table 1:** Baseline characteristics of the study population stratified by cognitive function.

Variable	Normal Cognition (*n* = 396)	Mild Cognitive Impairment (*n* = 303)	Severe Cognitive Impairment (*n* = 140)	*P*-value
Demographics				
Age (years)	64 (57, 69)	63 (58, 69)	63 (57.75, 68)	0.958
Sex (Male, *n* %)	270 (68.2%)	204 (67.3%)	103 (73.6%)	0.394
Height (cm)	168 (163, 173)	166 (160, 171)	163 (158, 168)	<0.001
Weight (kg)	72.0 (65.0, 80.0)	72.00 (65.0, 78.5)	69.50 (63.75, 75.0)	0.018
BMI (kg/m²)	25.82 (23.56, 27.59)	26.06 (24.22, 28.28)	25.91 (24.39, 27.47)	0.102
Education				0.267
Primary School & Below	84 (21.2%)	66 (21.8%)	19 (13.6%)	
Middle & High School	197 (49.7%)	156 (51.5%)	75 (53.6%)	
Higher Education	115 (29.0%)	81 (26.7%)	46 (32.9%)	
Lifestyle				
Smoking (*n*, %)	68 (17.2%)	46 (15.2%)	19 (13.6%)	0.558
Drinking (*n*, %)	41 (10.4%)	38 (12.5%)	17 (12.1%)	0.640
Clinical Characteristics				
Hypertension (*n*, %)	264 (66.7%)	190 (62.7%)	88 (62.9%)	0.496
Diabetes (*n*, %)	138 (34.8%)	124 (40.9%)	56 (40.0%)	0.222
SBP (mmHg)	129 (119, 139)	132 (118, 142)	134 (122, 145)	0.009
DBP (mmHg)	76 (69, 83)	76 (69, 83)	76.5 (68, 82.25)	0.988
TyG Index	8.66 (8.30, 8.99)	8.80 (8.48, 9.22)	8.89 (8.59, 9.27)	<0.001
Cognitive Scores				
MoCA Score	27 (27, 28)	24 (22, 25)	19 (15.75, 21.25)	<0.001
MMSE Score	29 (28, 30)	28 (28, 29)	24 (22, 26)	<0.001
Metabolic Indices				
Glu (mmol/L)	5.00 (4.49, 6.00)	5.32 (4.72, 6.99)	5.43 (4.81, 6.95)	<0.001
TG (mmol/L)	1.34 (1.02, 1.81)	1.47 (1.11, 1.94)	1.58 (1.17, 2.12)	<0.001
TC (mmol/L)	3.69 (3.14, 4.21)	3.63 (3.15, 4.42)	4.01 (3.25, 4.68)	0.025
HDL-C (mmol/L)	1.00 (0.88, 1.17)	0.99 (0.87, 1.13)	0.99 (0.84, 1.16)	0.454
LDL-C (mmol/L)	1.91 (1.48, 2.39)	1.88 (1.50, 2.46)	2.08 (1.55, 2.74)	0.086
nonHDL-C (mmol/L)	2.66 (2.07, 3.15)	2.63 (2.13, 3.33)	2.91 (2.28, 3.72)	0.011
RLP-C (mmol/L)	0.64 (0.50, 0.83)	0.70 (0.56, 0.89)	0.73 (0.59, 0.93)	<0.001
Liver/Kidney Function				
eGFR (mL/min)	91.23 (79.74, 100.13)	89.47 (78.39, 96.45)	85.16 (68.70, 93.98)	<0.001
Cr (μmol/L)	74.20 (66.07, 84.45)	73.70 (64.50, 84.50)	72.15 (60.52, 86.00)	0.189
Urea (mmol/L)	5.04 (4.17, 6.05)	5.24 (4.51, 6.25)	5.12 (4.31, 6.53)	0.034
UA (μmol/L)	322.50 (270.90, 379.93)	317.00 (267.35, 381.15)	313.55 (266.48, 383.27)	0.742
ALT (U/L)	17.00 (12.00, 23.00)	17.00 (13.00, 24.00)	15.00 (11.00, 21.00)	0.102
AST (U/L)	16.00 (13.00, 20.00)	16.00 (14.00, 21.00)	17.00 (14.00, 21.00)	0.118
GGT (U/L)	22.00 (16.00, 32.00)	23.00 (17.00, 35.00)	22.00 (17.00, 35.25)	0.200
ALP (U/L)	69.50 (58.00, 82.00)	75.00 (62.00, 87.50)	76.00 (66.00, 92.25)	<0.001
Alb (g/L)	42.10 (40.30, 44.30)	42.10 (40.20, 44.20)	41.05 (38.90, 43.42)	<0.001
A/G Ratio	1.67 (1.55, 1.85)	1.63 (1.49, 1.85)	1.58 (1.35, 1.78)	<0.001
T-Bil (μmol/L)	11.10 (8.76, 14.87)	11.43 (9.32, 14.47)	9.77 (7.00, 12.51)	<0.001
Other Indices				
hs-CRP (mg/L)	0.79 (0.47, 1.77)	0.84 (0.51, 2.18)	1.01 (0.57, 2.58)	0.009
Hcy (μmol/L)	13.00 (11.28, 15.10)	13.30 (11.40, 16.80)	13.80 (11.85, 18.05)	0.030
LD (U/L)	159.00 (144.00, 173.25)	158.00 (146.00, 181.00)	168.50 (148.75, 194.25)	0.001
*P* (mmol/L)	1.13 ± 0.17	1.17 ± 0.19	1.17 ± 0.18	0.004

Patients were stratified into three groups based on the concordance of screening test results: Normal Cognition: No impairment on either scale. Mild Cognitive Impairment (Discordant): Impairment on either MMSE or MoCA (predominantly isolated MoCA deficits), reflecting early or domain-specific decline.

Severe Cognitive Impairment (Concordant): Concurrent impairment on both MMSE and MoCA, reflecting advanced global decline. Data are presented as mean ± standard deviation (SD) for normally distributed continuous variables, median (interquartile range, IQR) for non-normally distributed variables, and number (percentage) for categorical variables. *P* values were calculated using One-way ANOVA for normally distributed data, Kruskal–Wallis H test for skewed data, and Chi-square test for categorical variables. A/G, albumin-to-globulin ratio; Alb, albumin; ALP, alkaline phosphatase; ALT, alanine aminotransferase; AST, aspartate aminotransferase; BMI, body mass index; Cr, creatinine; DBP, diastolic blood pressure; eGFR, estimated glomerular filtration rate; GGT, gamma-glutamyl transferase; Glu, fasting blood glucose; Hcy, homocysteine; HDL-C, high-density lipoprotein cholesterol; hs-CRP, high-sensitivity C-reactive protein; LD, lactate dehydrogenase; LDL-C, low-density lipoprotein cholesterol; MMSE, mini-mental state examination; MoCA, montreal cognitive assessment; nonHDL-C, non-high-density lipoprotein cholesterol; P, serum phosphorus; RLP-C, remnant lipoprotein cholesterol; SBP, systolic blood pressure; T-Bil, total bilirubin; TC, total cholesterol; TG, triglycerides; TyG, triglyceride-glucose index; UA, uric acid.

Stratification by TyG index tertiles (Table 2) revealed a progressive deterioration in metabolic health. Patients in the highest tertile (Tertile 3) presented with a pronounced dysmetabolic cluster, including significantly higher BMI, glucose, LDL-C, and hs-CRP levels, coupled with lower HDL-C. While hemodynamic parameters (blood pressure) and hypertension prevalence remained stable across groups, the burden of diabetes increased sharply with rising TyG levels. Crucially, a clear inverse gradient was observed between metabolic status and cognitive function; scores on both the MMSE and MoCA significantly declined as the TyG index increased, it indicates that a possible link exists between the degree of insulin resistance and impairments in cognitive function.

**Table 2:** Baseline characteristics stratified by tertiles of the TyG index.

Variable	T1 (7.37–8.53) (*n* = 283)	T2 (8.54–8.97) (*n* = 279)	T3 (8.98–10.93) (*n* = 277)	*P*-value
Demographics				
Age (years)	65 (58, 70)	63 (58, 68)	62 (56, 68)	0.063
Sex (Male, *n* %)	193 (68.2%)	198 (71.0%)	186 (67.1%)	0.604
Height (cm)	168 (162, 172)	167 (160, 172)	165 (159, 172)	0.073
Weight (kg)	71.0 (63.5, 77.0)	72.0 (65.0, 80.0)	72.0 (65.0, 80.0)	0.044
BMI (kg/m²)	25.22 (23.30, 27.31)	26.06 (24.23, 28.08)	26.57 (24.46, 28.40)	<0.001
Education				0.500
Primary School & Below	60 (21.2%)	56 (20.1%)	53 (19.1%)	
Middle & High School	133 (47.0%)	144 (51.6%)	151 (54.5%)	
Higher Education	90 (31.8%)	79 (28.3%)	73 (26.4%)	
Lifestyle				
Smoking (*n*, %)	46 (16.3%)	52 (18.6%)	35 (12.6%)	0.149
Drinking (*n*, %)	39 (13.8%)	31 (11.1%)	26 (9.4%)	0.258
Clinical Characteristics				
Hypertension (*n*, %)	179 (63.3%)	172 (61.6%)	191 (69.0%)	0.167
Diabetes (*n*, %)	76 (26.9%)	94 (33.7%)	148 (53.4%)	<0.001
SBP (mmHg)	129 (118, 139.5)	130 (120, 141)	133 (120, 142)	0.077
DBP (mmHg)	76 (68.5, 81)	76 (69, 83)	77 (69, 84)	0.393
Cognitive Function				<0.001
Normal	164 (58.0%)	129 (46.2%)	103 (37.2%)	
Mild Impairment	90 (31.8%)	100 (35.8%)	113 (40.8%)	
Severe Impairment	29 (10.2%)	50 (17.9%)	61 (22.0%)	
Cognitive Scores				
MoCA Score	26 (24, 28)	25 (23, 27)	24 (21, 27)	<0.001
MMSE Score	29 (28, 30)	29 (27, 30)	28 (26, 30)	<0.001
Metabolic Indices				
TyG Index	8.26 (8.10, 8.41)	8.76 (8.66, 8.86)	9.34 (9.12, 9.64)	<0.001
Glu (mmol/L)	4.69 (4.33, 5.12)	5.14 (4.62, 5.97)	6.95 (5.59, 10.26)	<0.001
TG (mmol/L)	1.01 (0.85, 1.18)	1.51 (1.29, 1.77)	2.12 (1.68, 2.67)	<0.001
TC (mmol/L)	3.36 (2.94, 3.91)	3.79 (3.25, 4.44)	4.01 (3.43, 4.74)	<0.001
HDL-C (mmol/L)	1.07 (0.93, 1.23)	0.99 (0.87, 1.13)	0.93 (0.82, 1.07)	<0.001
LDL-C (mmol/L)	1.72 (1.34, 2.18)	2.05 (1.59, 2.60)	2.04 (1.55, 2.69)	<0.001
nonHDL-C (mmol/L)	2.25 (1.81, 2.81)	2.75 (2.29, 3.38)	3.00 (2.45, 3.75)	<0.001
RLP-C (mmol/L)	0.52 (0.43, 0.65)	0.70 (0.58, 0.82)	0.91 (0.72, 1.08)	<0.001
Liver/Kidney Function				
eGFR (mL/min)	88.82 (78.14, 95.70)	91.15 (79.38, 99.44)	88.06 (74.94, 97.52)	0.036
Cr (μmol/L)	74.70 (66.20, 84.40)	73.70 (63.35, 84.10)	73.30 (63.80, 84.80)	0.215
Urea (mmol/L)	5.06 (4.27, 6.14)	4.99 (4.22, 5.99)	5.31 (4.53, 6.48)	0.005
UA (μmol/L)	310.40 (266.20, 353.15)	324.60 (269.90, 383.15)	335.20 (269.60, 400.90)	0.004
ALT (U/L)	15.00 (11.00, 20.00)	18.00 (13.00, 24.50)	18.00 (13.00, 25.00)	<0.001
AST (U/L)	16.00 (13.00, 19.00)	16.00 (14.00, 20.00)	17.00 (14.00, 22.00)	0.138
GGT (U/L)	19.00 (15.00, 26.00)	23.00 (17.00, 33.00)	27.00 (19.00, 41.00)	<0.001
ALP (U/L)	71.00 (60.00, 83.00)	72.00 (60.00, 85.00)	74.00 (62.00, 89.00)	0.053
Alb (g/L)	41.20 (39.60, 43.30)	42.30 (40.35, 44.30)	42.50 (40.40, 44.50)	<0.001
A/G Ratio	1.66 (1.51, 1.88)	1.65 (1.48, 1.81)	1.63 (1.46, 1.82)	0.106
T-Bil (μmol/L)	11.65 (8.77, 14.87)	10.92 (8.51, 14.41)	10.55 (8.48, 13.78)	0.066
Other Indices				
hs-CRP (mg/L)	0.68 (0.46, 1.30)	0.87 (0.51, 2.54)	1.09 (0.57, 2.67)	<0.001
Hcy (μmol/L)	13.20 (11.45, 16.75)	13.40 (11.20, 16.05)	13.20 (11.40, 16.20)	0.968
LD (U/L)	157.00 (143.00, 175.00)	160.00 (147.50, 179.50)	165.00 (145.00, 184.00)	0.026
*P* (mmol/L)	1.12 ± 0.18	1.16 ± 0.18	1.18 ± 0.18	<0.001

Abbreviations are the same as in Table 1.

### Correlation between TyG index and risk factors for cognitive dysfunction

**Figure 2:**
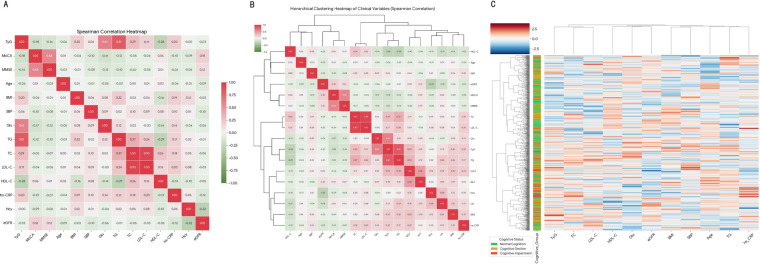
Correlation analysis and hierarchical clustering patterns linking the TyG index to cognitive dysfunction and metabolic phenotypes. **(A)** Spearman correlation heatmap of the TyG index, cognitive scores, and metabolic risk factors. Numbers represent correlation coefficients **(r)**. **(B)** Hierarchical clustering dendrogram identifying two distinct variable clusters: a “Metabolic-Inflammatory” cluster (TyG, TG, hs-CRP) and a “Cognitive-Protective” cluster (HDL-C, eGFR). **(C)** Two-way hierarchical clustering heatmap. The vertical axis clusters patients based on metabolic profiles, visualizing a distinct “Insulin-Resistant Phenotype” (high TyG, red density) that coincides with higher rates of cognitive impairment (indicated by the sidebar).

Correlation analysis (Figure 2A) demonstrated that the TyG index was positively correlated with BMI and hs-CRP, while inversely correlated with HDL-C. Hierarchical clustering (Figure 2B) segregated clinical variables into two opposing groups: a Metabolic-Inflammatory cluster (including TyG, TG, and hs-CRP) and a Cognitive-Protective cluster (including HDL-C and eGFR). Furthermore, the two-way heatmap (Figure 2C) identified distinct patient phenotypes. The Insulin-Resistant Phenotype, characterized by elevated TyG levels, coincided with a high density of cognitive decline and impairment, whereas the Metabolically Healthy Phenotype was predominantly associated with normal cognition. These findings confirm that the TyG index identifies a high-risk metabolic profile strongly linked to cognitive impairment.

### Association between TyG index and cognitive function

Multivariable logistic regression analysis demonstrated that the TyG index was independently associated with the risk of cognitive impairment (Table 3). In the fully adjusted model (Model 3), each 1-unit increment in the TyG index was associated with a 58.3% increase in the odds of mild cognitive impairment (OR: 1.583, 95% CI: 1.198–2.091, *P* = 0.001). The association was even more pronounced for severe cognitive impairment, with a more than two-fold increase in risk per unit rise in TyG (OR: 2.318, 95% CI: 1.583–3.394, *P* < 0.001). Categorical analysis confirmed these findings. Compared to the lowest tertile (T1), patients in the highest TyG tertile (T3) faced a significantly higher risk of both mild cognitive impairment (OR: 1.866, 95% CI: 1.268–2.748, *P* = 0.002) and severe cognitive impairment (OR: 3.255, 95% CI: 1.870–5.668, *P* < 0.001).

**Table 3:** Logistic regression models for the association between TyG index and cognitive function.

Outcome/TyG Tertile	Model 1 OR (95% CI)	*P*-value	Model 2 OR (95% CI)	*P*-value	Model 3 OR (95% CI)	*P*-value
Mild Cognitive Impairment (vs. Normal)						
TyG (continuous)	1.664 (1.275,2.171)	<0.001	1.663 (1.273,2.173)	<0.001	1.583 (1.198,2.091)	0.001
T1 (Ref)	1.000 (Reference)		1.000 (Reference)		1.000 (Reference)	
T2	1.413 (0.979,2.038)	0.065	1.411 (0.977,2.037)	0.066	1.382 (0.942,2.028)	0.098
T3	1.999 (1.380,2.896)	<0.001	2.001 (1.379,2.904)	<0.001	1.866 (1.268,2.748)	0.002
Severe Cognitive Impairment (vs. Normal)						
TyG (continuous)	2.217 (1.572,3.126)	<0.001	2.270 (1.602,3.217)	<0.001	2.318 (1.583,3.394)	<0.001
T1 (Ref)	1.000 (Reference)		1.000 (Reference)		1.000 (Reference)	
T2	2.192 (1.313,3.659)	0.003	2.222 (1.328,3.720)	0.002	2.202 (1.277,3.796)	0.005
T3	3.349 (2.019,5.556)	<0.001	3.431 (2.054,5.732)	<0.001	3.255 (1.870,5.668)	<0.001

Model 1: Unadjusted. Model 2: Adjusted for demographics (age, sex, education level). Model 3: Fully adjusted for demographics plus BMI, smoking, drinking, hypertension, LDL-C, eGFR, and hs-CRP. Odds ratios (ORs) represent the risk of cognitive impairment relative to the normal cognition group. CI, confidence interval; OR, odds ratio; TyG, triglyceride-glucose index.

**Figure 3:**
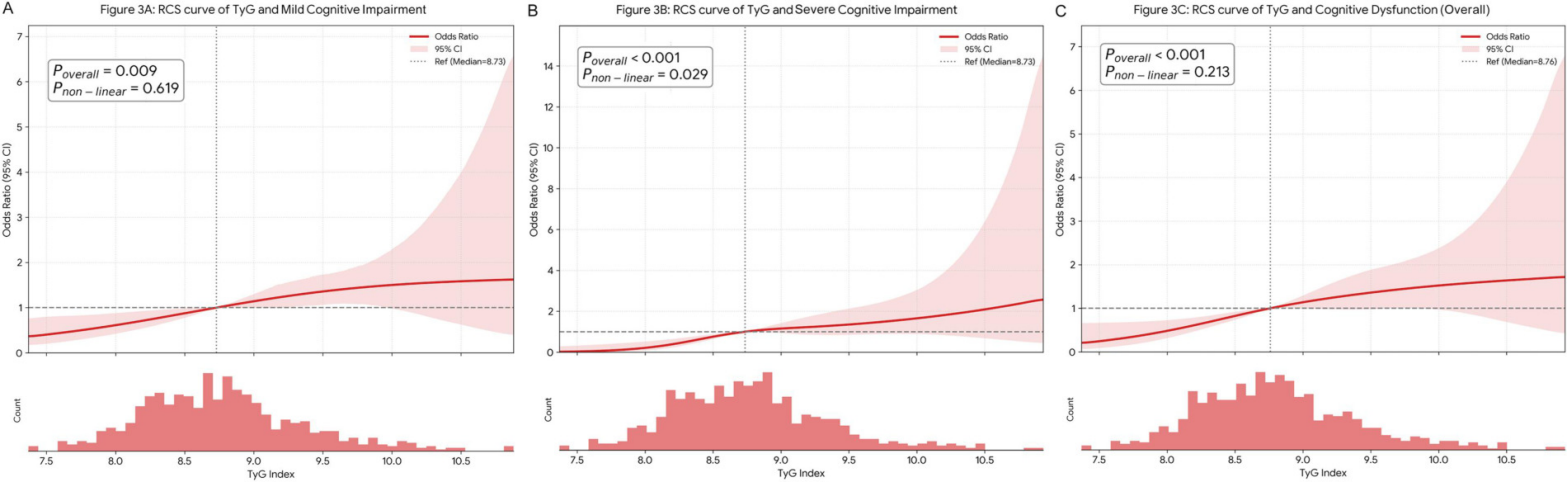
Restricted cubic spline analysis of the association between TyG index and cognitive outcomes. **(A)** Mild Cognitive Impairment, defined as discordant impairment (MoCA or MMSE < cut-off); **(B)** Severe Cognitive Impairment, defined as concordant impairment on both scales; and **(C)** Overall Cognitive Dysfunction. Models were adjusted for age, sex, education, BMI, smoking, drinking, hypertension, LDL-C, eGFR, and hs-CRP. Solid lines represent odds ratios (ORs) and shaded areas indicate 95% confidence intervals. The median TyG index (8.73) served as the reference. Note the distinct non-linear, J-shaped threshold effect observed specifically in the severe (concordant) impairment group.

Visualized via restricted cubic splines (Figure 3), these risk trajectories diverged significantly. While mild cognitive impairment followed a linear dose-response pattern (*P* for non-linearity = 0.619), severe cognitive impairment exhibited a distinct non-linear, J-shaped escalation (*P* for non-linearity = 0.029). This contrast indicates that while mild dysfunction is linearly associated with metabolic burden, severe neurocognitive impairment is predominantly observed at higher levels of insulin resistance, suggesting a potential threshold effect.

### Receiver operating characteristic (ROC) curve analysis and incremental effect of the TyG index for predicting cognitive dysfunction

**Figure 4:**
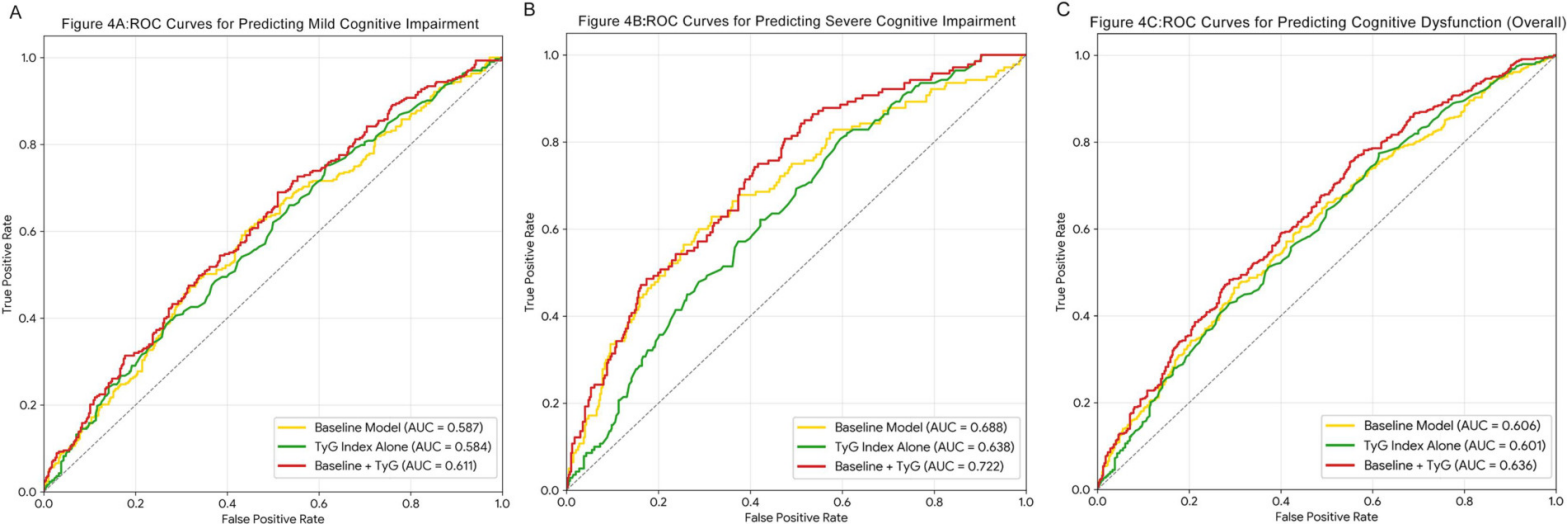
Receiver operating characteristic (ROC) curves evaluating the predictive value of the TyG index for cognitive outcomes. **(A)** Mild Cognitive Impairment; **(B)** Severe Cognitive Impairment; **(C)** Overall Cognitive Dysfunction. The blue line represents the baseline model (including age, sex, education level, BMI, smoking, drinking, hypertension, LDL-C, eGFR, and hs-CRP). The red line represents the combined model (Baseline Model + TyG index). The Area Under the Curve (AUC) values and *p*-values indicate the discriminatory performance of each model. ROC, receiver operating characteristic; AUC, area under the curve.

To quantify the prognostic utility of the TyG index beyond standard clinical covariates, we performed ROC curve analysis and reclassification statistics (Figure 4 and Table 4). The baseline risk model incorporated ten conventional factors, including demographics, lifestyle habits, and comorbidities. For mild cognitive impairment, adding the TyG index yielded a modest improvement in discrimination (AUC increased from 0.587 to 0.611) and a statistically significant NRI of 0.185 (*P* = 0.024). It's worth noting that the incremental predictive value was most pronounced for severe cognitive impairment. The inclusion of the TyG index significantly elevated the AUC from 0.688 to 0.722. Reclassification metrics further underscored this benefit, revealing a substantial improvement in risk stratification (NRI: 0.405, *P* < 0.001) and discrimination slope (IDI: 0.031, *P* = 0.023). These data indicate that the TyG index captures meaningful residual risk, significantly enhancing the identification of patients vulnerable to severe neurocognitive deficits.

**Table 4:** Incremental predictive value of the TyG index for predicting cognitive dysfunction.

Outcome	Model	NRI (95% CI)	*P*-value (NRI)	IDI (95% CI)	*P*-value (IDI)
Mild Cognitive Impairment	Baseline Model (Reference)	Ref	-	Ref	-
	Baseline Model + TyG Index	0.185 (0.024, 0.346)	0.024	0.015 (−0.004, 0.033)	0.117
Severe Cognitive Impairment	Baseline Model (Reference)	Ref	-	Ref	-
	Baseline Model + TyG Index	0.405 (0.200, 0.609)	<0.001	0.031 (0.004, 0.058)	0.023
Cognitive Dysfunction (Overall)	Baseline Model (Reference)	Ref	-	Ref	-
	Baseline Model + TyG Index	0.246 (0.089, 0.403)	0.002	0.023 (0.002, 0.043)	0.034

The baseline model included age, sex, education level, BMI, smoking, drinking, hypertension, LDL-C, eGFR, and hs-CRP. NRI, net reclassification improvement; IDI, integrated discrimination improvement; CI, confidence interval.

### Subgroup analyses

**Figure 5:**
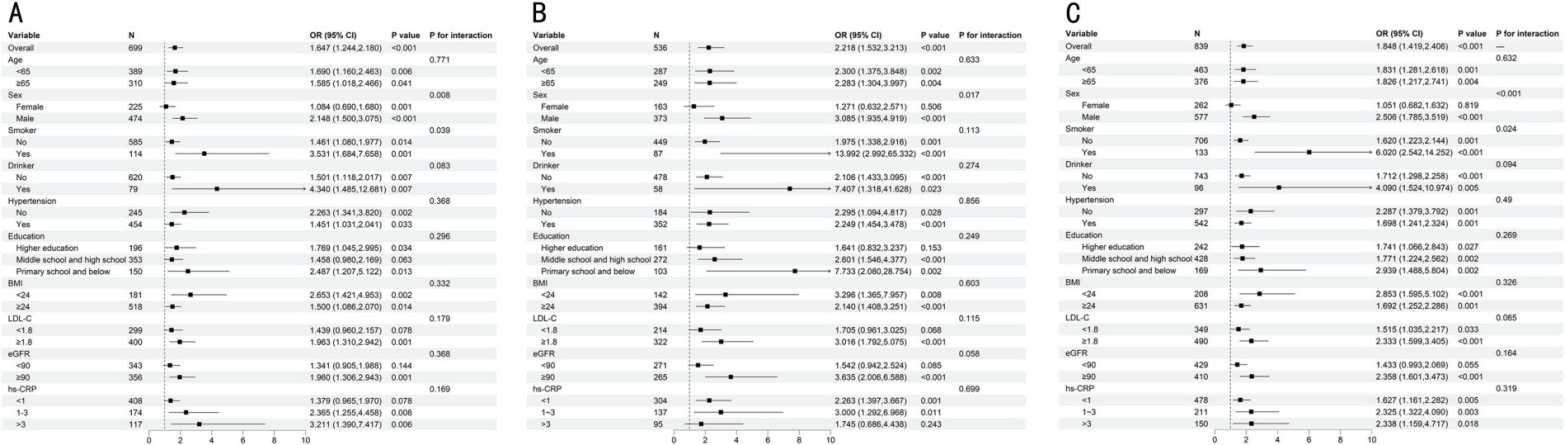
Forest plots evaluating the association between the TyG index and cognitive outcomes across various clinical subgroups. **(A)** Mild Cognitive Impairment; **(B)** Severe Cognitive Impairment; **(C)** Overall Cognitive Dysfunction. The forest plots display the odds ratios (ORs) and 95% confidence intervals (CIs) derived from the fully adjusted logistic regression model. In each subgroup analysis, the model was adjusted for age, sex, education level, BMI, smoking, drinking, hypertension, LDL-C, eGFR, and hs-CRP, except for the variable used for stratification. Wide confidence intervals in the smoker subgroup indicate limited precision due to sample size. OR, odds ratio; CI, confidence interval.

Subgroup analyses stratified by key clinical characteristics evaluated the consistency of the association between the TyG index and cognitive outcomes (Figure 5). While the deleterious impact of elevated TyG levels was evident across most subpopulations—including stratifications by age, BMI, and hypertension—significant effect modification was observed for sex and smoking status.

Specifically, the association was markedly stronger in male participants compared to females across all cognitive phenotypes. The disparity was most pronounced in the overall cognitive dysfunction model, where males exhibited a significantly higher risk estimate (OR: 2.506, 95% CI: 1.785–3.519) than females (OR: 1.051, 95% CI: 0.682–1.632; *P* for interaction < 0.001). Similar patterns were observed for mild (Male OR: 2.148 vs. Female OR: 1.084; *P* for interaction = 0.008) and severe cognitive impairment (Male OR: 3.085 vs. Female OR: 1.271; *P* for interaction = 0.017). Additionally, smoking status significantly modified the risk, with active smokers exhibiting much higher risk estimates than non-smokers for both mild cognitive impairment (OR: 3.531, 95% CI: 1.684–7.658 vs. OR: 1.461, 95% CI: 1.080–1.977; *P* for interaction = 0.039) and overall dysfunction (OR: 6.020, 95% CI: 2.542–14.252 vs. OR: 1.620, 95% CI: 1.223–2.144; *P* for interaction = 0.024). Despite this statistical heterogeneity, the directionality of the risk remained largely positive, suggesting the TyG index as a broadly applicable risk marker, albeit with heightened sensitivity in men and smokers.

## Discussion

In this cross-sectional analysis of participants with established CHD, we identified the TyG index as an independent and graded predictor of cognitive function. Beyond a univariate association, our hierarchical clustering analysis identifies a specific clinical profile characterized by the confluence of insulin resistance, dyslipidemia, and systemic inflammation. This trait means having high TyG, high triglycerides, and systemic inflammation all grouped together, and it's strongly linked to cognitive function. An important finding of this study is the differential dose-response pattern: a linear trajectory for mild cognitive impairment contrasting with a threshold-dependent, J-shaped association for severe cognitive impairment. Furthermore, the substantial net reclassification improvement (NRI > 0.40) observed when adding the TyG index to standard cardiovascular risk models suggests that conventional stratification, which predominantly focuses on hemodynamic and LDL-dependent pathways, fails to capture the significant residual neurocognitive risk conferred by IR in the cardiovascular population (20–22).

The strong association observed in our cohort must be contextualized within the specific pathophysiology of the “heart-brain axis”. Patients with coronary disease are already characterized by a pre-existing pathological substrate of chronic cerebral hypoperfusion and endothelial rarefaction, resulting from generalized atherosclerosis and reduced cardiac output (23, 24). In this vulnerable neurovascular environment, our findings suggest that systemic insulin resistance, quantified by the TyG index, plays a critical role in further damage. Unlike the general population, where the cognitive impairment of metabolic dysregulation is typically delayed until late life (25), the brain of a CHD patient operates with diminished vascular reserve. In this precarious state, the impairment of neurovascular coupling—a hallmark of insulin resistance—becomes clinically manifest much earlier. Insulin receptors are densely expressed in the hippocampus and frontal cortex, areas pivotal for memory and executive function (26). Under normal conditions, insulin signaling regulates synaptic plasticity and clearance of amyloid-beta (27, 28). We propose that high TyG levels may reflect a state of central insulin resistance where these protective mechanisms are blunted, exacerbating the neuronal injury caused by chronic ischemia.

A key distinction of the TyG index compared to other markers like fasting glucose or HbA1c is its incorporation of the lipid component. Our results highlight that the cognitive risk is likely driven by the synergistic toxicity of glucose and triglycerides—a phenomenon termed “glucolipotoxicity” (29). While the brain is traditionally considered an insulin-independent glucose consumer, recent evidence has challenged the oversimplification of this view. It is now recognized that insulin receptors are widely expressed in brain microvascular endothelial cells and neurons, and insulin signaling plays a critical role in maintaining blood-brain barrier (BBB) integrity (30). Consistent with this, hypertriglyceridemia—an integral component of the TyG index—has been shown to impair BBB function: excess triglycerides are hydrolyzed into free fatty acids (FFAs), which induce oxidative stress and inflammatory responses in endothelial cells, thereby disrupting the tight junctions of the BBB (31). Excess FFAs, often elevated in parallel with triglycerides, can cross the compromised BBB in CHD patients, inducing neuroinflammation and oxidative stress within the parenchyma (32, 33). The hierarchical clustering analysis in our study (Figure 2), which grouped TyG closely with hs-CRP rather than with renal or hemodynamic markers, supports the notion that this is not merely a metabolic defect but an inflammatory one. This “neurotoxic triad” of hyperglycemia, lipid overload, and inflammation likely accelerates the transformation of microglia into a pro-inflammatory M1 phenotype, promoting aberrant synaptic pruning and neuronal apoptosis (34, 35). This mechanism may explain why the TyG index outperformed traditional lipid markers (like LDL-C) in predicting cognitive outcomes in our models; LDL particles do not cross the BBB as readily as triglycerides and free fatty acids do.

A notable finding of this study is the distinct dose-response relationship: a linear trend for mild impairment vs. a J-shaped curve for severe impairment. This divergence aligns with the “Metabolic Reserve Hypothesis”. In the earlier stages of insulin resistance, corresponding to lower TyG levels, the brain likely maintains function through compensatory mechanisms, characterized by an adaptive metabolic shift toward utilizing alternative substrates, such as lactate and ketone bodies (36, 37). Thus, mild TyG elevations result in proportional risk increases. However, the highest TyG tertile likely represents a saturation point where these adaptive reserves become exhausted. Beyond this threshold, the failure of metabolic flexibility may accelerate mitochondrial stress and neuronal dysfunction (38, 39), driving the non-linear progression to severe cognitive deficits. These results have practical clinical implications, suggesting that preventing patients from crossing this specific high-risk threshold may be as important as general risk factor management.

Our subgroup analyses provided further granular insights, revealing a significant interaction with sex and smoking status. The observation that the TyG-cognitive association was markedly stronger in men warrants specific attention. Biologically, this may stem from differences in adipose tissue distribution; men typically exhibit greater visceral adiposity for a given BMI compared to women, who tend to store fat subcutaneously (40). Visceral adipose tissue is more metabolically active and proinflammatory, secreting higher levels of cytokines that can penetrate the central nervous system (41, 42). Consequently, a similar elevation in TyG index in a male patient may reflect a more “toxic” systemic inflammatory milieu than in a female patient. Similarly, the amplified risk observed in smokers suggests a synergistic interaction between oxidative stress induced by cigarette smoke and metabolic dysregulation. Smoking is known to damage endothelial integrity and increase BBB permeability, potentially facilitating the entry of neurotoxic lipids and inflammatory mediators associated with high TyG levels (43, 44). These interactions underscore the necessity for a personalized approach to risk stratification, where male smokers with elevated TyG levels are flagged as an “ultra-high risk” phenotype for cognitive deterioration.

Current guidelines for CHD management vigorously target LDL-C and blood pressure, yet a significant proportion of patients continue to develop cognitive decline. Our reclassification analysis indicates that the TyG index captures a substantial component of “residual risk” that remains unaddressed by statins and antihypertensives (45, 46). It is noteworthy that our multivariate models were adjusted for LDL-C, suggesting the risk conferred by TyG is independent of the cholesterol-centric pathway. This aligns with the concept that “residual cardiovascular risk” is largely metabolic and inflammatory in nature. In clinical practice, a patient with controlled LDL-C but an elevated TyG index is often considered “at goal” by traditional standards, yet our data classify them as high-risk for neurodegeneration, particularly for the severe impairment trajectory. Identifying these discordant phenotypes is crucial. The TyG index allows for the stratification of CHD patients who might superficially appear managed but are metabolically deteriorating, providing a rationale for shifting focus from purely hemodynamic goals to comprehensive metabolic neuroprotection.

Finally, our findings open specific avenues for therapeutic intervention. If the association is indeed causal, as the strong biological plausibility suggests, then agents that specifically improve insulin sensitivity and reduce triglyceride-rich lipoproteins may offer dual cardioprotective and neuroprotective benefits. The specific phenotype identified—high TyG without necessary overt diabetes—points towards the potential utility of insulin-sensitizing agents such as PPAR-gamma agonists (e.g., pioglitazone) or the newer GLP-1 receptor agonists and sodium-glucose cotransporter-2 (SGLT2) inhibitors. These drug classes have shown promise in improving endothelial function and reducing neuroinflammation (47–49).

Our study provides the rationale for future randomized controlled trials to specifically test whether targeting TyG reduction in non-diabetic CHD patients can decelerate the trajectory of cognitive decline. Until such data are available, the TyG index serves as an accessible, cost-effective “vital sign” for brain health in the cardiology clinic, prompting early lifestyle and pharmacological strategies to preserve cognitive reserve in this high-risk population.

## Strengths and limitations

A primary strength of this investigation is the detailed assessment of the dose-response relationship between insulin resistance and cognitive status. By utilizing restricted cubic splines rather than treating cognitive impairment as a binary outcome, we identified a distinctive threshold-dependent association for severe impairment. Additionally, the application of hierarchical clustering allowed us to visualize a specific “metabolic-inflammatory” phenotype, offering mechanistic insight into how the TyG index captures residual risk beyond traditional hemodynamic factors.

However, several methodological limitations warrant consideration: First, the cross-sectional design inherently precludes causal inference. While our findings suggest that metabolic dysregulation accelerates neurodegeneration, we cannot rule out reverse causality, where cognitive decline leads to poorer self-care and subsequent metabolic deterioration. Longitudinal validation is required to determine if TyG trajectories predict conversion rates to dementia.

Second, although adjusted for multiple confounders, residual confounding remains a concern. Our definition of smoking and drinking was limited to status at admission, potentially missing the cumulative vascular burden of former smokers and drinkers. Similarly, while we adjusted for LDL-C levels, we lacked detailed data on the intensity and duration of statin therapy. Given that statins may exert pleiotropic effects on insulin sensitivity (50–52), this unmeasured variable could modulate the observed associations. The TyG index relies on fasting glucose, which may be modulated by antidiabetic medications in the 35%–40% of our cohort with diabetes. We did not consider the effect of glucose lowering on TyG values. In addition, in Table 1, the cognitively impaired population was significantly shorter than the normal population, differences in height are suggestive of impaired early development associated with poor childhood nutrition or lower socioeconomic status. Although we adjusted for education, this unmeasured early adversity may lead to residual confounding, which in turn affects cognitive function.

Third, our cognitive assessment relied on screening instruments (MMSE and MoCA) rather than a comprehensive neuropsychological battery. While this limits diagnostic precision, our use of a “concordance-based” severity classification—distinguishing between discordant impairment (one test failed; likely domain-specific or early vascular injury) and concordant impairment (both tests failed; likely global cortical involvement)—allowed for a clinically relevant stratification of risk. Future studies using domain-specific testing are needed to validate this grading system.

Finally, this study was conducted at a single tertiary center involving a homogenous Chinese population. While this reduces genetic confounding regarding adiposity profiles, it limits the generalizability of our findings to other ethnicities with different body composition phenotypes. Consequently, these results should be extrapolated with caution until validated in multi-ethnic cohorts.

## Conclusion

In patients with CHD, the TyG index acts as an independent predictor of cognitive dysfunction, showing a particularly strong association with severe impairment. Our data suggest that a specific metabolic-inflammatory phenotype—combining insulin resistance, dyslipidemia, and systemic inflammation—may accelerate neurodegeneration via pathways distinct from traditional hemodynamic risks. Notably, the non-linear, threshold-dependent relationship observed here explains why the TyG index improves risk stratification beyond standard models. These findings support the use of the TyG index to identify patients with residual metabolic risk who might benefit from therapies targeting insulin sensitivity and inflammation.

## Data Availability

The raw data supporting the conclusions of this article will be made available by the authors, without undue reservation.
